# Alveolar macrophages in lung cancer: opportunities and challenges

**DOI:** 10.3389/fimmu.2023.1268939

**Published:** 2023-09-26

**Authors:** Cheng-Yen Chang, Dominique Armstrong, David B. Corry, Farrah Kheradmand

**Affiliations:** ^1^ Department of Medicine, Baylor College of Medicine, Houston, TX, United States; ^2^ Department of Pathology and Immunology, Baylor College of Medicine, Houston, TX, United States; ^3^ Biology of Inflammation Center, Baylor College of Medicine, Houston, TX, United States; ^4^ Center for Translational Research on Inflammatory Diseases, Michael E. DeBakey Department of Veterans Affairs Medical Center, Houston, TX, United States

**Keywords:** tissue-resident alveolar macrophages, smoking, lung cancer, immune checkpoint inhibitors, tumor-associated macrophages, tumor microenvironment

## Abstract

Alveolar macrophages (AMs) are critical components of the innate defense mechanism in the lung. Nestled tightly within the alveoli, AMs, derived from the yolk-sac or bone marrow, can phagocytose foreign particles, defend the host against pathogens, recycle surfactant, and promptly respond to inhaled noxious stimuli. The behavior of AMs is tightly dependent on the environmental cues whereby infection, chronic inflammation, and associated metabolic changes can repolarize their effector functions in the lungs. Several factors within the tumor microenvironment can re-educate AMs, resulting in tumor growth, and reducing immune checkpoint inhibitors (ICIs) efficacy in patients treated for non-small cell lung cancer (NSCLC). The plasticity of AMs and their critical function in altering tumor responses to ICIs make them a desirable target in lung cancer treatment. New strategies have been developed to target AMs in solid tumors reprograming their suppressive function and boosting the efficacy of ICIs. Here, we review the phenotypic and functional changes in AMs in response to sterile inflammation and in NSCLC that could be critical in tumor growth and metastasis. Opportunities in altering AMs’ function include harnessing their potential function in trained immunity, a concept borrowed from memory response to infections, which could be explored therapeutically in managing lung cancer treatment.

## 1 Introduction

Alveolar macrophages (AMs) are critical in maintaining lung homeostasis under normal and diseased conditions. In response to minimal irritants, AMs initiate low-grade inflammation followed by a resolution phase inducing immune tolerance, thus limiting the extent of lung injury. Under a static state, AMs express critical regulatory factors such as interleukin (IL)-10, the canonical anti-inflammatory cytokine (1), and express cell surface receptors including program cell death-ligand 1 (PD-L1) to optimize phagocytosis and prevent excessive inflammation in the lung (2). Severe tissue-damage disrupts the AM pool, primarily composed of tissue-resident AMs (TRAMs), which can be replenished by monocyte-derived AMs (MoAMs) (3, 4). In response to infection, AMs phagocytose infectious agents, which triggers secretion of pro-inflammatory cytokines and chemokines, activating the downstream adaptive immune response (5). Sterile inflammation, which occurs in the absence of microorganisms, can trigger a similar response (5). Chronic inflammation, infectious or sterile, leads to aberrant tissue repair signals and immune responses. The development of lung cancer is initiated and perpetuated by both extrinsic chronic inflammation and intrinsic DNA damage in the lungs related to cigarette smoke. Impaired AMs have been found in heavy smokers, showing deficiencies in antigen recognition and phagocytosis (6).

Antitumor functions of AMs have been described in non-small cell lung cancer (NSCLC) (7), but once integrated within solid tumors, they become a component of a heterogenous class of tumor-associated macrophages (TAMs). TAMs can induce exhausted T cells, promote immune suppression, and reduce the efficacy of ICIs in NSCLC (8). Therefore, several strategies have been developed to target TAMs, aiming to resolve their function in inducing a suppressive microenvironment. These efforts aim to increase T cell trafficking in solid tumors, thus improving the efficacy of ICIs.

NSCLCs account for around 85% of cancer cases and include adeno (40%), squamous (25%), and large cell (10%) carcinoma subtypes (9). Despite differences in histology, early-stage NSCLC is amenable to resection and carries a favorable prognosis (10). However, over 70% of cancer cases are diagnosed at advanced stages (11), and carry a poor prognosis (12). In the past decade, the incorporation of ICIs, targeting cytotoxic T lymphocyte-associated protein 4 (CTLA4), program cell death 1 (PD-1), and PD-L1 have significantly advanced the treatment paradigm in NSCLC. For patients with tumor PD-L1 expression of > 50%, ICIs are the first line treatment choice showing a promising overall survival rate (12). However, the overall response rate remains low at approximately 20% in non-selected NSCLC (13), indicating the need to improve reactivating the exhausted tumor-specific T cells.

The tumor microenvironment (TME) shapes immune cells’ fate and governs treatment outcomes (14). Specifically, tumors have evolved to sustain energy, compete for local resources, evade immune cell surveillance, and thrive under extreme conditions making them highly adaptive to changes in TME (15). A critical mechanism responsible for tumor evasion includes altering macrophage function, which are abundant in the lung TME (16), by disabling their antitumor function while licensing them to increase tolerance. Consistently, increase in macrophage density within TME correlates negatively with patient survival in several types of solid tumors, including NSCLC (17). Tumor cells can further recruit and polarize monocytes accelerating growth/angiogenic factor production, increasing proteinases, and facilitating tumor invasion, suggesting co-evolution between these two cell types (18).

**Figure 1:**
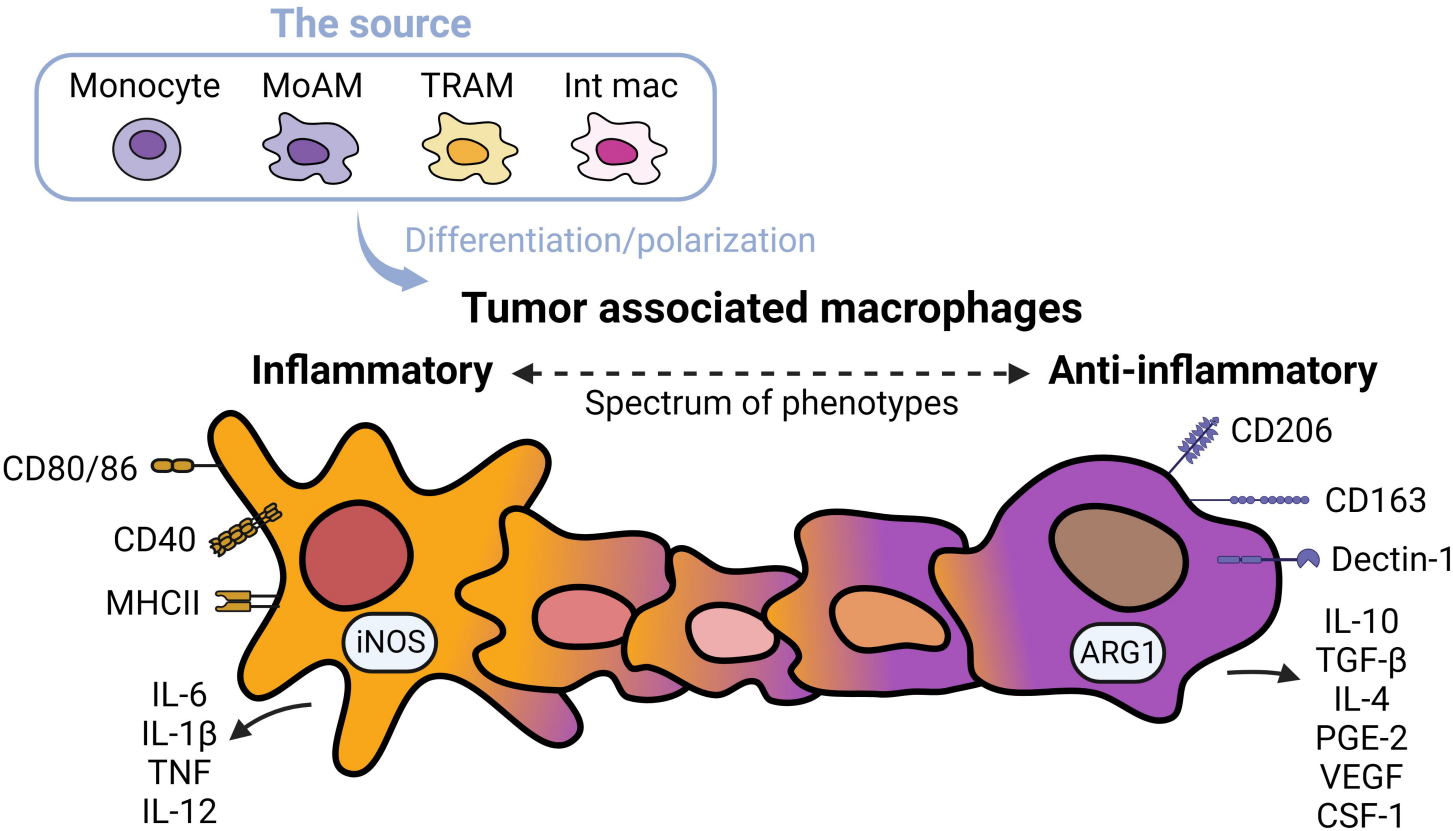
Spectrums of tumor associated macrophages. Tumor associated macrophages (TAMs) can develop from monocytes, monocyte-derived alveolar macrophages (MoAMs), tissue-resident alveolar macrophages (TRAMs), or interstitial macrophages (IMs) in the lung. TAMs exist as a spectrum of phenotypes from inflammatory to anti-inflammatory cells. Inflammatory macrophages are defined by higher expression of antigen-presenting and co-stimulatory molecules including CD80/CD86, CD40, MHCII, and secretion of inflammatory cytokines, whereas anti-inflammatory macrophages are immunosuppressive, expressing CD163 and Dectin-1, higher levels of CD206, and secretion of immunosuppressive cytokines and angiogenic factors. Typically, anti-inflammatory TAMs are more abundant in NSCLC.

TAMs promote tumor progression and metastasis in several murine models of NSCLC (19). TAMs represent heterogenous populations which exist on a spectrum of phenotypes from inflammatory to anti-inflammatory macrophages (**Figure 1**) (20). Markers of inflammatory macrophages, major histocompatibility complex (MHC) class II co-stimulatory molecules (CD40, CD80, and CD86), and markers of anti-inflammatory macrophages (CD163 and CD206) have been detected in TAMs in early-stage NSCLC (21). TAMs are often considered anti-inflammatory-skewed macrophages because of their immunosuppressive phenotypes (22); however, the exact expression of inflammatory/anti-inflammatory macrophage phenotype in humans is less clear. Chronic inflammation associated with TAMs is linked to malignant cell transformation (23). For instance, inflammatory TAMs accelerate genome instability, whereas anti-inflammatory TAMs express immunosuppressive molecules facilitating tumor progression (24). Anti-inflammatory TAMs directly inhibit T and natural killer (NK) functions by engaging between checkpoint ligands and receptors. Alternatively, the secretion of immunosuppressive cytokines, exosomes, metabolites, and immunosuppressive enzymes also inhibits anti-tumor immunity and fosters tumor growth (25).

The critical roles of AMs in lung cancer have prompted focused studies on identifying their ontogeny and lineages. Although the ontogeny of AMs in humans is undefined, murine TRAMs can self-renew, whereas monocyte-derived macrophages primarily repopulate from hematopoietic stem cells and accumulate in inflamed regions (26). AMs detect foreign substances in the airways, defend the host from pathogens, and recycle lipids (e.g., surfactant). AMs also interpret lung signals from the environmental, leading to rapid and reversible changes in their function (27). However, chronic inflammation polarizes and impairs AMs’ function (28, 29). Here we review relevant discoveries related to AMs in chronic inflammation and in NSCLC. We discuss the regulation of PD-1/PD-L1 in AMs and their potential reprogramming as a novel therapeutic option for NSCLC.

## 2 Origin and function of alveolar macrophages

### 2.1 Development and origin of AMs

**Figure 2:**
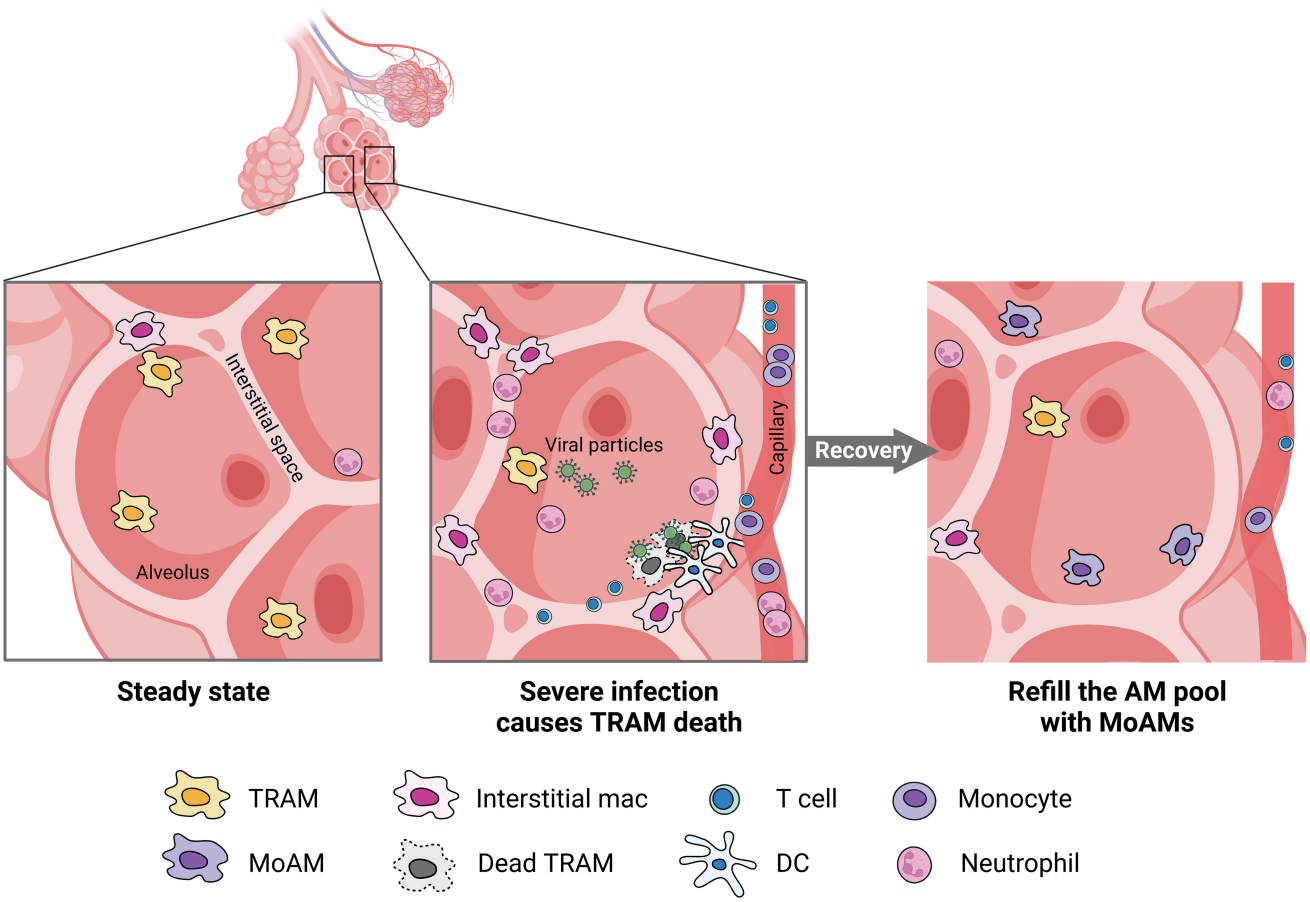
MoAMs replace TRAMs after severe lung infection. Severe infection leads to the death of TRAMs but attracts monocytes, neutrophils, DC, and T cells from the blood stream to the alveolar space. Increased interstitial macrophages can be found in the lungs. After recovery, MoAMs replace the loss of TRAMs in the alveoli.

AMs encompass both TRAMs and MoAMs. Advanced technology and lineage tracing has increasingly enabled the ability to distinguish these populations in mice, but less is known in humans. In mice, TRAMs are derived from yoke sac precursors of fetal monocytes that populate the alveoli shortly after birth and persist over the lifespan. The precursors are a self-renewing, embryo-derived population and are independent of bone marrow contribution (30). TRAMs rely on the granulocyte-macrophage colony-stimulating factor (GM-CSF) for their function (31). TRAM precursors in humans are not well-defined but the process may be similar across species because defects in GM-CSF production or auto-antibodies against GM-CSF disrupts TRAMs function causing alveolar proteinosis in mice and humans (32). GM-CSF is also critical for peroxisome proliferator-activated receptor-γ (PPARγ) expression, a ligand-activated nuclear receptor, required for TRAM differentiation (33). PPARγ is constitutively expressed in TRAMs under steady states and is required for the alveolar lipid homeostasis (34). The autocrine activation of TGF-β can also trigger PPARγ expression (35). Notably, in mice TRAMs can locally restore a moderately depleted pool without the need for recruiting MoAMs. However, in response to severe lung injury or an overwhelming infection, TRAM repletion requires MoAMs to engraft the AM pool (**Figure 2**) (3, 4). There is some evidence that over the course of natural aging, monocyte infiltrate and differentiate into AMs in the lungs (30). Although in early stages of inflammation, MoAMs can be readily separated from TRAMs, recruited monocytes progressively acquire similar functions, but retain specific genes linked to the circulatory origin (36). The functional differences between these two origins of cells, and their effect on tissue responses to injury, remain less clear.

Lineage tracing including the membrane-spanning 4-domains subfamily A member 3 (Ms4a3), found in granulocyte-monocyte and common monocyte progenitors has been used to study macrophages (37). Ms4a3 reporter and Cre fate-mapping models track monocyte-derived macrophages and granulocytes but not dendritic cells (DCs) or embryonic tissue-resident macrophages in the tissue (38), thereby distinguishing MoAMs from mature TRAMs. Another fate-mapping model used to specify TRAMs from the hematopoietic origin of macrophages is membrane-associated protein 17 (MAP17) reporter, which marks adult hematopoietic stem cells (39). However, a critical caveat is that monocyte fate-mapping models using *Cx3cr1^Cre^* or *Cx3cr1^CreERT2^* also label DC, and *LyzM^Cre^* labels other myeloid cells, restricting their use in understanding TRAMs source (37). Future research is needed to determine whether TRAMs and MoAMs are functionally redundant or whether they have distinct roles in tissue repair, infection, and cancer.

### 3.2 Function of AMs in the lungs under steady state

**Figure 3:**
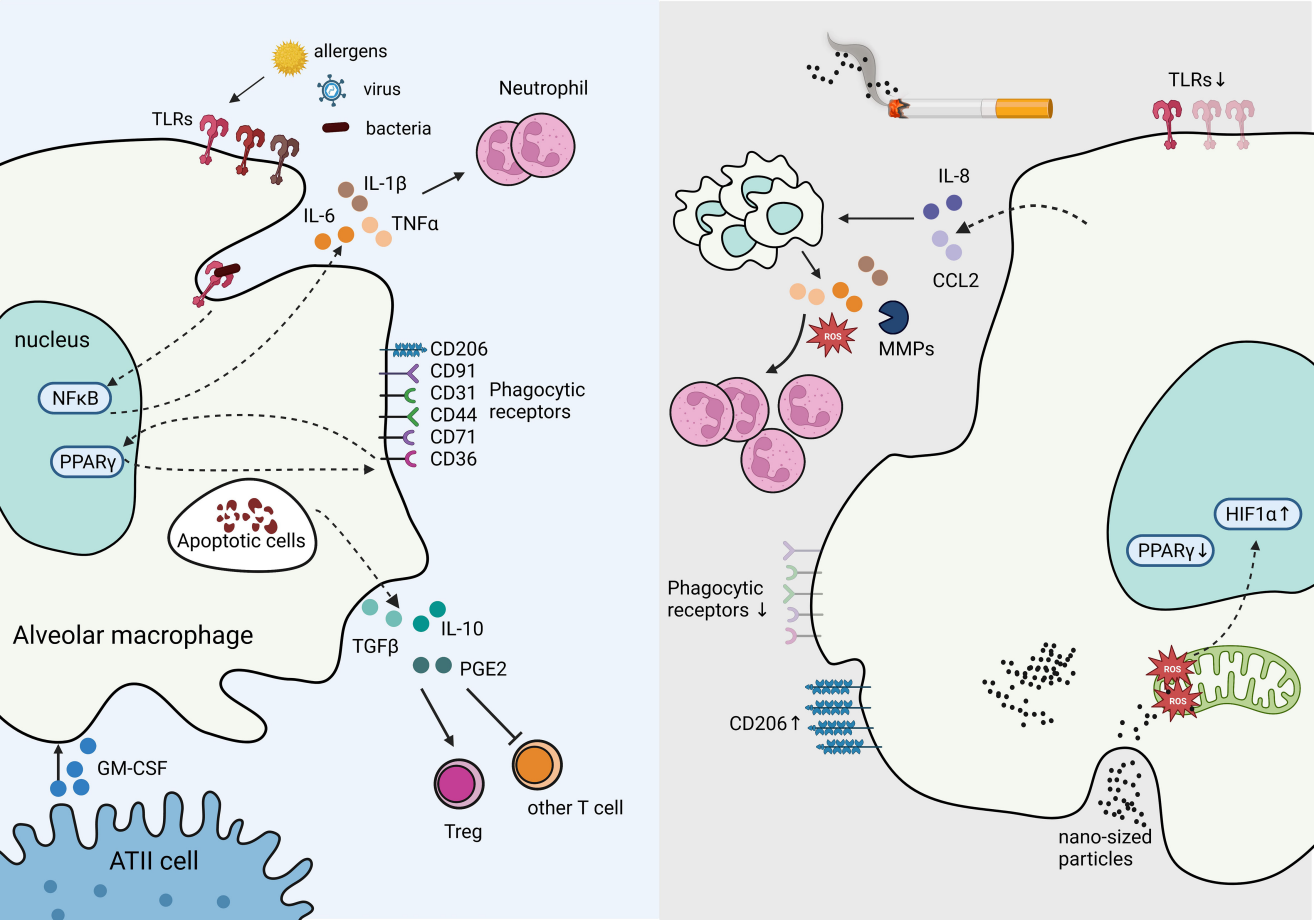
Tissue Resident Alveolar Macrophages in homeostasis and immune suppression in response to chronic inflammation. The production of GM-CSF from ATII cells supports AM survival, proliferation, and differentiation. Under homeostasis, AMs express high levels of pathogen recognition receptors (PRRs), such as toll-like receptors (TLRs) to rapid response to bacterial, and viral insults. Engagement of TLRs induces NFκB activation which stimulates inflammatory cytokines IL-6 and IL-1β production, and recruit neutrophils. Phagocytosing apoptotic cells stimulates suppressive signals IL-10, TGF-β, and PGE2 secretion mitigating tissue damage and inflammation. In contrast, cigarette smoke inhibits TLRs and phagocytic receptors dampening responses to viral and bacterial threats in AMs, while increasing CD206. Cigarette smoke stimulates IL-8 and CCL2 production, which recruits additional macrophages primed to secrete excessive ROS, MMPs, and other inflammatory cytokines, resulting in excessive neutrophils and causing tissue damage. Cigarette smoke generates nano-sized carbon which accumulates in AMs and penetrates mitochondria, inducing ROS and HIF-1α while decreasing PPARγ signaling.

AMs are capable of presenting antigens to T lymphocytes; however, under steady state, their low-level expression of co-stimulatory and MHC class II molecules reduces their ability to activate adaptive immunity (27). In contrast, in response to a high expression of damage-associated molecular patterns (DAMPs) and pathogen-associated molecular patterns (PAMPs) such as viral particles, AMs are poised to immediately respond to the threat (40). These responses include secreting pro-inflammatory cytokines IL-6, IL-1β, and tumor necrotic factor (TNF), propagating the innate immune responses (41). AMs are also essential in phagocytosing apoptotic cells (e.g., neutrophils) and toxic particles to prevent further tissue injury and leakage of immunogenic molecules from dead or dying cells, serving as a critical function in resolving inflammation (40). Phagocytosing apoptotic cells, known as efferocytosis, reprograms AMs to increase the expression of transforming growth factor-β (TGF-β), IL-10, and prostaglandin E2 (PGE2), abrogating the inflammatory responses (42). TGF-β further inhibits T lymphocyte activation and promotes regulatory T cells (Treg) differentiation (43), thus restraining activation of adaptive immune responses (**Figure 3**).

## 3 Chronic cigarette smoke and sterile inflammation impairs AM function

### 3.1 Smoking increases trafficking but impairs AM function

Chronic exposure to particulate matter, generated from incomplete combustion of organic matter (e.g., cigarette smoke, forest fires, environmental pollutants, etc.), causes sterile inflammation in the lungs and plays a key role in the activation of adaptive immunity in the lung (44). Cellular profiling of bronchial alveolar lavage (BAL) fluid in cigarette smokers show increased number of AMs, but their immune function is dampened as evidenced by downregulation of interferon-γ (IFN-γ) signaling and increased risk of lung infection (45). The paracrine and/or autocrine effects of IL-8 and CCL2 (also known as CXCL8 and MCP-1, respectively), induced by cigarette smoke are responsible for increased AM trafficking in the lungs (46). AMs secrete pro-inflammatory cytokines, matrix metalloproteinases (MMPs), and reactive oxygen species (ROS), leading to a degradation of extracellular matrix, oxidative stress, excessive recruitment of neutrophils and T helper (Th)17 cells, and tissue damage (28).

Smoking also reduces the expression of many cell surface recognition molecules in AM, including CD31, CD91, CD44, and CD71 and dampens their phagocytic activity (6). Toll-like receptor (TLR) downregulation has been shown in AMs after exposure to cigarette smoke, further diminishing their antimicrobial function (47). Consistently, smoking can reduce AM’s ability to phagocytose fungal pathogen, *Candida albicans*, and gram-negative bacteria, *Klebsiella pneumoniae*, in murine models (48). Smoking can reduce the expression of inflammatory phenotypic markers and polarize AMs to an anti-inflammatory-like phenotype (49). Further, dual polarization of AMs has been reported in smokers with Chronic obstructive pulmonary disease (COPD), a chronic inflammatory lung condition that blocks pulmonary airflow, whereby inflammatory and anti-inflammatory macrophage markers are increased at the same time and correlate with disease severity (50). Interestingly, CD206 is an anti-inflammatory marker that is highly expressed in resting AMs (51) but is amplified in smokers (50) and after exposure to particulates (28), indicating a feedback mechanism that may be critical in resolving inflammation. Though increased expression of anti-inflammatory markers suggest an immune suppressive response, cytokine profile and functional assessments are required to determine the exact role of AMs in specific contexts.

### 3.2 Changes in metabolism alters AM phenotype and function

AMs are capable of intrinsic metabolic switching. Specifically, under steady-state or resting conditions, AMs preferentially use mitochondrial oxidative phosphorylation (OXPHOS), fueled by fatty acid and glucose oxidation to generate ATP (52). Once activated, AMs rapidly shift their metabolism and increase glycolysis to provide a large supply of ATP that further polarizes them toward a pro-inflammatory macrophage. Notably, in response to influenza infection, and in contrast to monocyte-derived macrophages, TRAMs stabilize hypoxia-inducible factor 1 α (HIF-1α), a process that is required for altering their metabolic responses (53). The accessory functions of AMs in the lung include lipid metabolism and homeostasis of surfactant, a complex of phospholipids and proteins that maintains pulmonary compliance and requires constitutive expression of PPARγ (34). The nuclear receptor transcription factor, PPARγ in AMs inhibits the expression of pro-inflammatory cytokines while enhancing phagocytic function (54). Other transcriptional genetic control of AMs includes mitochondrial transcription factor A (TFAM), whereby its deficiency results in impaired OXPHOS, leading to reduced numbers of mature AM, accumulated surfactants, and increased susceptibility to infection (55).

Chronic exposure to cigarette smoke dysregulates AM metabolism (56). Smokers show disrupted redox homeostasis, reduced PPARγ, and increased nitric oxide and ROS production in mitochondria, with reduced antioxidant glutathione, exacerbating lung inflammation (57). Increased ROS is also found in AMs after phagocytosing nanotubes and nano-particulate carbon black, a byproduct of smoking (28). In contrast, PPARγ plays a protective role against oxidative stress by transcriptional repression of pro-inflammatory factors and enzymes, such as NF-κB, induced nitric oxide synthase (iNOS), and the activation of antioxidant genes, such as heme oxygenase-1 (HO-1) and superoxide dismutase (SOD) (58). PPARγ also participates in glucose and lipid metabolism (54), and regulates the scavenger receptor, CD36, to control the surfactant lipid catabolism (59). Current smokers show decreased CD36 in AMs compared to former smokers (60), indicating that smoking may impair surfactant lipid catabolism and increase NF-κB activation (61) through deregulated surfactant recycling. Notably, smoke-induced reduction of lipid metabolism and PPARγ signaling in AMs is independent of COPD phenotype (62). Furthermore, smoking-associated AM clusters show upregulated genes involved in detoxification, inflammation, and oxidative stress that strengthen COPD progression (62). Additionally, transferrin receptor, CD71, is downregulated in smokers, permitting inappropriate pulmonary bacterial growth and pulmonary fibrosis (47, 63). CD71 expression in mature AMs can limit free iron available to be used by pathogenic microorganisms. AMs lacking CD71 exhibit reduced maturation, phagocytic function, and increased profibrotic genes (64). Advanced COPD lungs are enriched with high metallothionein-expressing AMs, indicating disrupted heavy metal metabolism (60). Although metallothionein has protective roles in acute lung injury (65), there is limited mechanistic information about elevated metallothionein in advanced COPD.

## 4 The role of AMs in NSCLC

### 4.1 AM polarization

**Figure 4:**
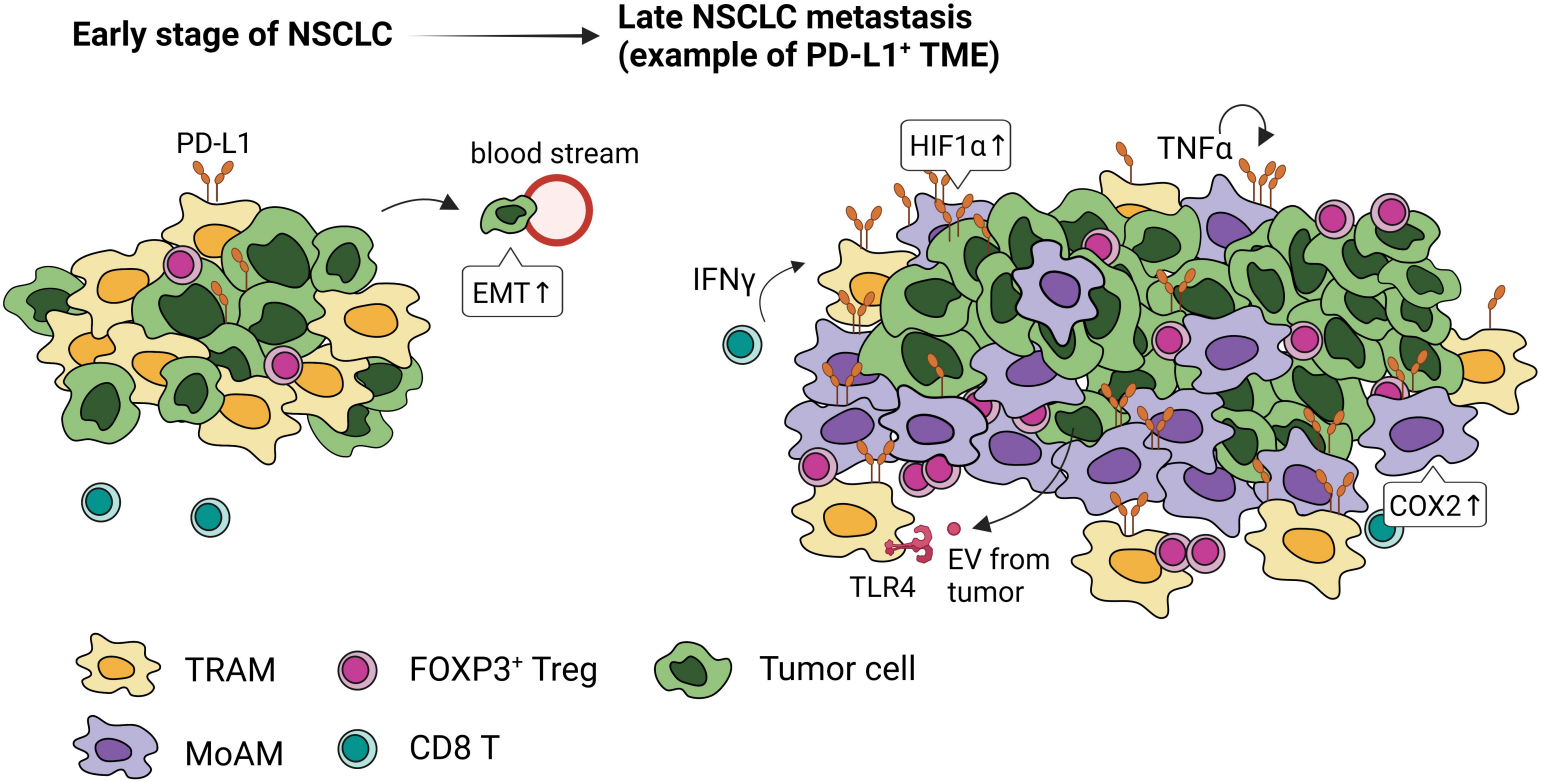
PD-L1 expression and localization of macrophages in early and late stages of NSCLC. Tumor-associated macrophages express high levels of PD-L1 through activation of HIF1α, COX2 signaling, paracrine IFN-γ, autocrine TNFα, and extracellular vesicles (EVs) from tumor cells. In the early stage of NSCLC, embryonic-origin TRAMs are adjacent to the tumor cells. They surround the tumor mass preventing CD8 T cell infiltration, promoting FOXP3^+^Tregs, and inducing epithelial-mesenchymal transition of tumor cells. In the late stage of NSCLC, these TRAMs are in the periphery and MoAMs dominate the tumor.

Both the promotion and inhibition of lung tumor have been attributed to AMs. AMs isolated from lung cancer patients when stimulated with IFN-γ or GM-CSF, secrete TNF-α, IL-6, and IL-1β, that can enhance tumor killing (66). However, mounting evidence suggest that many antitumor function of AMs, such as phagocytosis and the expression of cytokines that activate adaptive immunity, wane with the progression of NSCLC (67), pointing to their role in promoting immunosuppressive microenvironment and dysfunctional immunity (**Figure 4**).

Acute inflammation induces DC maturation, required for antigen presentation, and initiation of anti-tumor responses from effector T cells. In contrast, chronic inflammation, a hallmark of advanced NSCLC (68), promotes suppressive environment hindering the efficacy of immunotherapy, exacerbating cancer development (69). Chronic inflammatory mediators recruit TAM to tumors sites inhibiting T cell function and facilitating tumor invasion. While some bone marrow-derived Ly6C^+^ monocytes express CCR2 in lung tumors other immunosuppressive monocytic myeloid-derived suppressor cells (M-MDSC) can also differentiate into TAM upon exposure to hypoxia (70). TAMs are highly heterogenous in nature and in addition to hematopoietic origin (37), include polarized AMs (71). Although MoAMs and TRAMs make up TAMs population in human NSCLC, their functional differences, surface marker expression, gene regulation, and cell fate, remain largely unknown.

### 4.2 TRAMs foster lung cancer

TRAMs are highly represented within TAMs in NSCLC (39, 72). Single cell (sc)RNA sequencing and lineage tracing studies have identified distinct temporal and spatial distribution of TAMs in mouse and human lung (39). In the early-stage NSCLC, TRAMs are highly proximal to tumors; however, MoAMs gradually replace TRAMs and infiltrate the advanced NSCLC lesions (39) (**Figure 4**). TRAMs upregulate genes involved in antigen presentation and tissue remodeling, promoting epithelial mesenchymal transition and tumor invasion (39). Depletion of TRAMs before tumor engraftment can lead to lower tumor burden, reduced FOXP3^+^ Tregs, and increased IFN-γ^+^ CD8 T cells, indicating TRAMs support the early stage of NSCLC development (39).

Targeted molecular therapy for epidermal growth factor receptor (EGFR) mutation has improved patients’ responses; however, nearly all tumors develop resistance after treatment (73). Animal models of EGFR-driven NSCLC have shown that AMs are critical in the development of primary lung adenocarcinoma. Using an inducible knock-in model that mimics the development of mutant *EGFR* lung lepidic adenocarcinoma in humans, the oncogenic signaling led to a massive expansion of AMs with an immunosuppressive phenotype (8, 74). Similar results have been reported in the *Kras*; *p53* mouse model of NSCLC (75). AMs upregulate IL-1R and phagocytosis while downregulating MHCII and co-stimulatory molecules, consistent with an anti-inflammatory phenotype (76). Specific elimination of AMs but not interstitial macrophages (IMs) drastically reduced tumor burden (74, 76) and increased cytotoxic CD8 T cell infiltration in the lung (74). When compared to early and late invasive lung adenocarcinoma, studies have shown that AMs (defined as CD11c^+^ F4/80^+^ CD64^+^ Siglec-F^+^) are replaced by TAMs (CD11b^+^ F4/80^+^ CD64^+^ Siglec-F^-^) as cancer progresses (8). Why and how AMs are progressively replaced by monocyte-derived macrophages in the TAM pool during tumor expansion remains to be determined.

TRAMs play a pivotal role in promoting immunosuppression in the metastatic niches. In a model of metastatic breast cancer, TRAMs accumulate in the premetastatic lung area, a process that was dependent on complement C5a receptor and local production of C5 (72). TRAMs inhibited lung DC maturation by promoting Th2 cell generation and reducing tumoricidal activity (72). Depleting TRAMs reversed the immune suppression in the lungs, strengthened Th1 responses, and reduced metastatic burden (72). Notably, bone marrow transplant experiments showed that the transplanted cells did not become AMs in the lung; instead, most of them become MDSCs, expressing CD11b and Gr-1, excluding the contribution of bone marrow to the TRAM pool (72). In the metastatic lung tumor, foamy TRAMs were shown to express the lipid metabolic receptors *Lgals3* and *Trem2* and inhibit T cell effector function (77). Similarly, in a metastatic hepatocellular carcinoma model, TRAMs exhibited a high level of the inflammatory eicosanoid derivative leukotriene B4 (LTB4), by expressing 5-lipoxygenases (5-LOX), enabling cancer cell proliferation (63). CCR2^+^ MoAMs are recruited by CCL2^+^ interstitial macrophages in the metastatic foci, indicating a systemic signal repletes AM pool from bone marrow (63). TRAM depletion decreased metastatic foci numbers, demonstrating a direct role of TRAMs in lung metastasis (63). These studies show that distinct signals from the primary tumor can manipulate TRAMs to promote metastasis support the pool by a variety of mechanisms; however, the actual signal molecules or mediators that come from the primary tumor are still largely unknown.

### 4.3 Regulation of PD-L1 expression in AMs

The interaction between PD-1 on tumor-infiltrating lymphocytes (TILs) and PD-L1 on tumor cells is a critical mechanism for immune escape and most widely targeted with ICI. Although many solid tumors express PD-L1, immune cells including CD68^+^ TAMs, exhibit high levels of PD-L1 (78) (**Figure 4**) and some cases, PD-1 (8). Notably, ICI-treated NSCLC patients with elevated PD-L1^+^ macrophages have a more favorable survival than those with high PD-L1 in tumor cells (78). Further, a high level of PD-L1^+^ macrophages positively correlate with PD-L1/PD-1 expression in tumor cells and TILs, respectively (78). The single-cell analysis combined with spatial quantification identified clusters of PD-L1^+^ macrophages at the tumor-invasive margin, posing a physical barrier that can block T cell entry in the tumor lesion (79). The efficacy of ICI-mediated antitumor responses depends on whether TILs can recognize and kill tumor cells; therefore, increased TIL infiltration indicates “hot tumors” and carries favorable treatment outcome (80). PD-L1^+^ TAMs that hinder effector T cell function are commonly described in syngenetic tumor transplant and advanced models of solid tumors (81, 82). However, in humans PD-L1^+^ TAMs in early-stage of NSCLC do not directly inhibit T cell responses (83), highlighting the complex biology of human tumor development. Some of the differences between animal models and human tumors include tumor latency with selective pressures and delayed immune infiltration occurring in the later stages of cancer (84). TRAMs are a critical innate immune player, and their phenotypes are dictated by the surrounding TME in NSCLC. Under static conditions, they constitutively express PD-L1 to increase their phagocytosis and repress the effector T cell activation (2). However, whether and how TRAMs alter PD-L1 expression at different stages of tumor development in humans could provide critical information in predicting those who may benefit from PD-L1/PD-1-based immunotherapy.

Tissue hypoxia, a microenvironmental factor that induces polarization and PD-L1 expression in TAMs, has been shown to promote NSCLC progression (85). HIF-1α upregulates PD-L1 in TAMs, thereby supporting an immunosuppressive TME (86). Combination therapies based on HIF-1α inhibition and PD-1/PD-L1 checkpoint blockade have been shown to induce tumor regression, alleviate immune suppression, and increase survival in a murine model of NSCLC (87). Although specific changes in TAMs were not examined, small-molecule inhibition of HIF-1α promoted antitumor immunity, indicating HIF1α inhibition may be a promising adjuvant with ICI treatment (87).

Cytokines and soluble factors within the TME, including IFN-γ, TNF, COX2, and TLRs, can also induce PD-L1 expression in TAMs. Evidence has shown that the inflammatory cytokines IFN-γ from tumor-specific T cells can drive PD-L1 expression in tumors and surrounding stroma that expresses interferon receptors (88). While endogenous IFN-γ is dispensable for AMs’ PD-L1 expression, intrinsic TNF is required for its maintenance and upregulation (89). The inflammatory COX2/mPGES1/PGE2 pathway contributes to PD-L1 expression in tumor-infiltrating myeloid cells (81). The anti-inflammatory cytokine IL-10 has been reported to increase TAM PD-L1 in several cancers (90). Notably, tumor-derived extracellular vesicles captured by TLR4 in macrophages activate STAT3-dependent PD-L1 transcription, which polarizes them to anti-inflammatory phenotype (91). Together, PD-L1 expression by TAM through paracrine and autocrine signaling emphasizes the multifaceted and complex interactions that occur in the TME. Thus, further studies are needed to more comprehensively delineate the upstream stimulations that induce PD-L1^+^ TAM to advance the development of therapeutic intervention and treatment regimes.

### 4.4 Factors Responsible for Trained Immunity in AMs

Metabolic reprogramming and epigenetic imprinting drive innate immune memory responses, also known as trained immunity. AMs responding to pathogens, can protect the host from subsequent related or unrelated microbial exposure (92). Whether TRAMs or MoAMs develop trained immunity is context dependent. TRAMs can increase MHC-II, defense-related genes, glycolysis, and chemokine expression after adenovirus infection. This priming requires contact with IFN-γ^+^ CD8 T cells (93). In contrast, TRAMs depleted by influenza infection are replaced by MoAMs, which confer protection post-influenza infection against other microbial pathogens (94). These recruited MoAMs show similar surface markers of TRAMs but with a transcriptional profile that resembles CCR2^+^ monocyte precursors, and open chromatin at loci controlling the expression of inflammatory genes (94). However, MoAM production of IL-6 wanes 2 months post-influenza and protection against bacteria is lost. Interestingly, TRAMs become unresponsive, with poor phagocytic ability after the resolution of systemic inflammation, a process that is dependent on signal-regulatory protein α (SIRPα) (95). In pneumoniae, LPS-mediated induction of trained AMs adoptively transferred to naïve mice and challenged with *Streptococcus pneumoniae*, failed to protect mice against the infection, indicating niche environments are critical for triggering effective trained immunity (96). These findings suggest that infection severity and the microenvironment can determine whether trained or tolerant AMs are generated.

Less is known about the role of trained macrophages in tumor immunity. Trained macrophages can develop from systemic hematopoietic progenitors and circulating monocytes or TRAMs (97, 98). Antitumor responses in lungs have been described in mice recovered from acute influenza infection, indicating that trained AMs may play a pivotal role in determining the fate of tumor development. Trained AMs can increase phagocytosis, promote cytotoxicity, and resist tumor-induced immune suppression, showing distinct transcriptomic and epigenetic profiles (99). Notably, this trained immunity is dependent on IFN-γ and NK cells (99). Different routes of antigen exposure can train independent pools of macrophages. For example, in the metastatic setting, beta-glucan particles *via* i.p. has been shown to result in trained interstitial macrophages but not AMs (100). Future research should specify which populations of macrophages are affected by stimulation and what environmental signals are involved in the trained immunity.

### 4.5 Targeting TAMs to treat NSCLC

**Figure 5:**
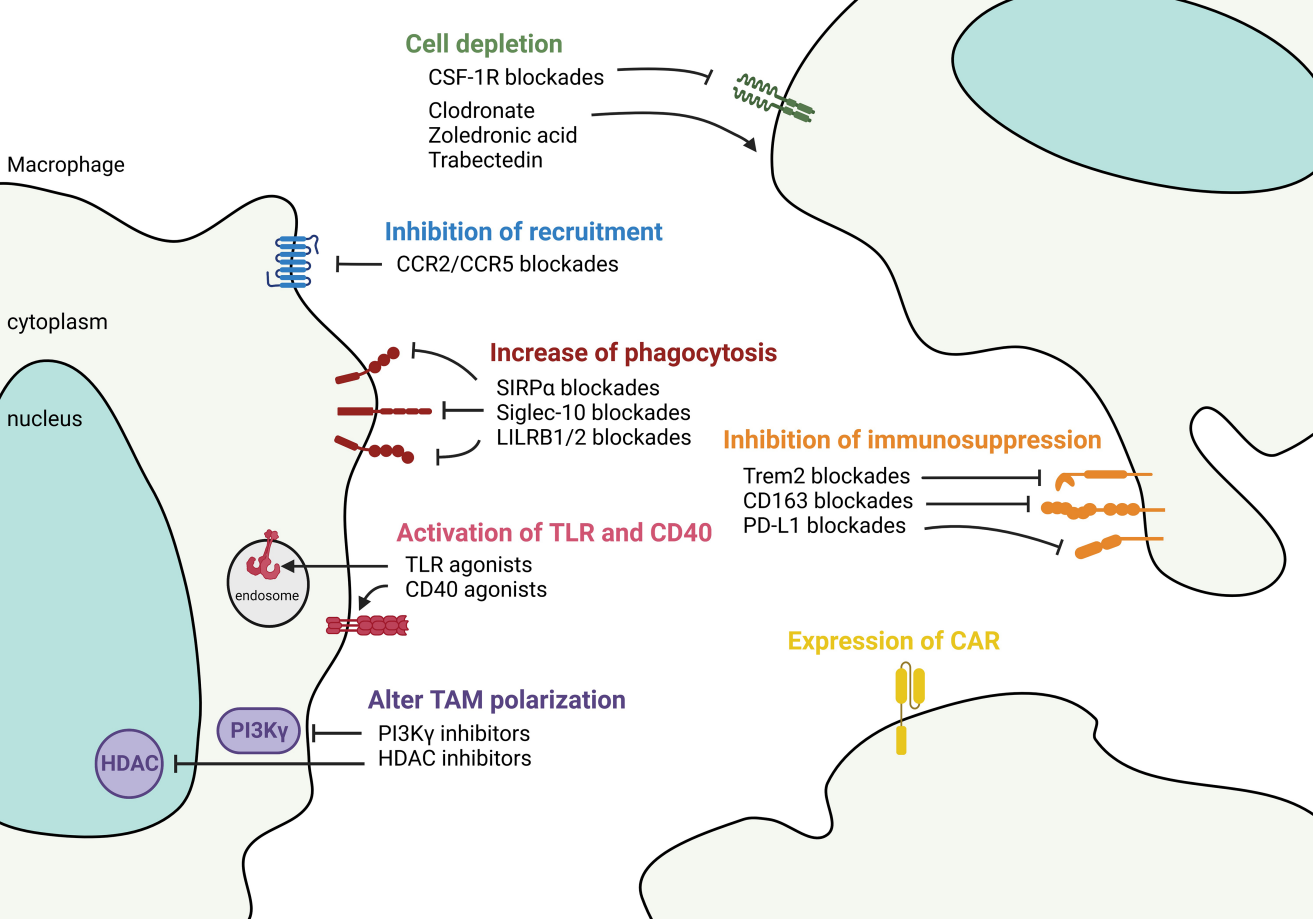
Targeting macrophages to control tumor microenvironment. Therapeutic strategies aimed at controlling TAM in the TME fall into 1) controlling the number of TAMs either by anti-CSF-1R and chemicals such as clodronate, zoledronic acid, and Trabectedin to deplete the cells, or by chemokine blockades to decrease the recruitment of TAMs. 2) Functional-based reprogramming that targets the surface receptors/ligands. For example, increase phagocytosis by SIRPα, Siglec-10, and LILRB1/2 blockades; activate TLR and CD40 signaling by agonists; inhibit immunosuppression by targeting Trem2, CD163, and PD-L1. 3) Pan reprogramming that inhibits the intracellular key regulators such as PI3Kγ and HDAC to prevent TAM polarization. 4) Expression of engineered CAR in TAMs to recognize and phagocytose targeted tumor cells.

A recent trend to control tumor growth is to deplete, block, or reactivate TAMs and synergistically activate T cells in the TME. This is accomplished by depleting or stimulating TAMs to become inflammatory macrophages, concurrently with ICI treatment to awaken tumor-specific T cell responses. Several reviews have discussed this topic in solid tumors (101, 102), therefore, here we provide an update of this information, focusing on targeting TAMs in NSCLC (**Figure 5**).

Depletion of TAM by inducing cell death can inhibit tumor growth. Both MoAMs and TRAMs express CSF-1R (103). Targeting the CSF-1/CSF-1R pathway can deplete monocytes/macrophages in animal models (104). However, antibody-based CSF-1R blockade alone has only a modest antitumor effect in humans (105). Combining the anti-CSF-1R with ICI in murine models led to significant tumor reduction (106), however, systemic depletion of macrophages either causes undesired side effects (107) or insufficient antitumor response in NSCLC patients (108, 109). A small study that compared the spatial transcriptomic profiles in NSCLC tumors suggested that combined anti-CSF-1R with ICI may not be clinically beneficial because ICI responders showed higher expression of CSF-1R (110). This finding underlines the heterogeneity of TAMs and beneficial roles of macrophage subsets in the TME. Similarly, another study showed that anti-CSF-1R can deplete macrophages but have a limited effect on antitumor responses in mice bearing established tumors (111). Despite limited clinical activities, CSF-1R kinase inhibitors combing with other drugs are currently in phase I trials for advanced solid tumors. Small molecules that mimic bone matrix pyrophosphatases, including clodronate and zoledronic acid, and Trabectedin that induces DNA damage can deplete macrophages (112). More studies are needed to decipher whether TAM depletion combined with other agents could provide clinical applications.

A combination of blacking monocyte recruitment in patients with ICI therapy represents a potential new treatment strategy in NSCLC. Several small molecule inhibitors targeting chemokine receptors can decrease monocyte-derived macrophage recruitment to NSCLC. Targeting CCL2/CCR2 and CCL5/CCR5 pathways have shown promising pre-clinical results in NSCLC (113), and have been expanded to clinical trials in adult solid tumors (NCT04504942 and NCT04123379). The CCL2/CCR2 axis attracts TAMs through PD-1 signaling in esophageal carcinogenesis (114). CCL5 from macrophages facilitates colorectal cancer growth *via* PD-L1 stabilization (115). A caveat is that once bone marrow-derived macrophages enter the tumor, the expression of chemokine receptors may be downregulated, as has been shown in ovarian cancer, where CCR2 expression is regulated by tumor-derived TNF (116). Also, TRAMs play a pivotal role in cancer initiation similarly express a lower level of chemokine receptors; therefore, targeting chemokine pathways may not be able to remove these cells.

Reprogramming TAMs at the tumor site is a newly emerging strategy for cancer treatment. “Don’t eat me” axis, e.g., CD47/SIRPα and CD24/Siglec-10 between tumor cells and myeloid cells presents an exciting paradigm for using tumor-induced inhibitory pathways to escape immune surveillance. Specifically, CD47 is commonly overexpressed in solid tumors where it is recognized by macrophages’ SIRPα, preventing phagocytosis (117). NSCLCs co-expressing CD47 and PD-L1 are associated with worse clinical outcomes (118). Blocking the interaction between CD47 and macrophage SIRPα increases cytosolic DNA sensing and activates the STING pathway activation that enables detection of tumor mitochondrial DNA and subsequent antigen presentation to T cells (119). More research is needed to better understand the mechanistic effects of blocking CD47/SIRPα in the TME. The first human anti-CD47 phase I trial showed that the drug is well tolerated in advanced tumors (120). Several CD47 blockades used as a monotherapy or combined with ICI or other agents are currently in clinical trials. Another “don’t eat me” signal, the recently discovered CD24/Siglec-10 axis facilitates macrophage infiltration into the tumor by Siglec-10-mediated sensing of CD24 and is complementary to CD47 signaling. Targeting both axes showed an additive phagocytosis response (121). Similarly, the disruption of the MHCI/LILRB1/2 axis potentiates phagocytosis by TAMs (122).

Several new approaches have shown that reprograming TAMs’ intracellular signaling can effectively alter their function. For instance, phosphoinositide 3 kinase gamma (PI3Kγ), a family member of PI3K, controls the switch between immune stimulating or suppressive macrophages (123). PI3Kγ is highly expressed on myeloid cells but not on cancer cells and mediates myeloid cell trafficking in cancer (123). PI3Kγ blockade stimulates inflammatory macrophages, enhances effector CD8 T cell function, and increases ICI responses in tumors (123). The PI3K/AKT/mTOR pathway is critical in NSCLC development. Studies have shown that inhibition of the epigenetic regulator, histone deacetylase (HDAC), can transcriptionally modify TAMs and activate their antitumor activity (124). The class IIa HDAC inhibitor (TMP195) has been shown in a metastatic breast cancer model to induce recruitment and differentiation of phagocytic macrophages, reducing primary tumor burden and incidence of lung metastasis (125). Combination with ICI further enhances the anti-tumor effect and durability of this HDAC IIa inhibition (125). Adjuvant therapy targeting epigenetic modulators (DNA methyltransferase and HDAC) after surgical removal of the tumor also showed inhibition of MDSC accumulation in the lung premetastatic niche and longer disease-free survival (126).

Activation through TLR signaling and CD40 can polarize TAMs to a pro-inflammatory state. This signaling converts tumors from cold to hot, activates the immune system, and overcomes ICI resistance. TLRs regulate TAM phenotypes, DC maturation, and increase effector T cell response (127). The anti-tumor property has led to the expansion of TLR agonists for immunotherapy. The use of TLR agonists in cancer is controversial because TLRs are also expressed on cancer cells and have pro- as well as anti-tumorigenic activities. However, several TLR agonists targeting TLR 3, 7, 8, and 9 are currently in trials for solid tumors (127). TLR agonists paired with ICI have shown synergistic effects on therapeutic efficacy and survival rate in patients (128). Future work is needed to understand the mechanistic regulation between TLR agonists and immune checkpoint expression in the TME. Similarly, CD40 is a potent agent to promote a TAM-mediated antitumor response. Combining CD40 agonist, anti-CSF-1R, and anti-PD-L1 in trio treatment has been evaluated in the MC38 syngeneic model and showed 90% complete response (129). The results of the phase I trial of this trio regimen in advanced tumors, which included NSCLC, showed no overt safety issue (130). Given the safety profile and antitumor activity, more clinical trials regarding these agonists with other therapeutic approaches in diverse solid tumors are warranted and anticipated.

Counteracting the immunosuppressive TME by targeting cell-surface suppressive markers on TAM to potentiate the efficacy of ICI is of growing interest. Triggering receptor expressed on myeloid cells 2 (TREM2) has been found on tumor cells, TAMs, and myeloid cells. In addition to its role in oncogenic signaling (131), TREM2^+^ TAM derived from monocytes strongly suppress NK cell function and promoting antitumor immunity in NSCLC (132). TREM2^+^ monocytes-derived macrophages have been found exclusively induced by the TME (133), and inhibit production of IL-18 and IL-15, which are necessary for NK activity (132). *Trem2* deficiency or anti-Trem2 treatment improved ICI responses and reduces tumor burden (134). Interestingly, two types of APOE^+^ TAMs are found, expressing either TREM2 or FOLR2 in human breast cancers. According to spatial analyses, TREM2^+^ macrophages are inside the tumor nest and close to the invasive margin, whereas FOLR2^+^ macrophages reside away from the tumor nest and remain in the perivascular area (135). FOLR2^+^ macrophage-CD8 T cell clusters correlate with a better clinical outcome (135). However, an in-depth characterization of macrophages and determination of their role in NSCLC is needed for their prognostic utilization. Another suppressive target is the scavenger receptor CD163 that positively correlates with tumor progression (136). PD-1^+^ TAMs in human NSCLC express CD163 and are associated with reduced survival (137). Interestingly, CD163^+^ C33^+^ PD-L1^+^ macrophages were retrospectively found to be higher prior to ICI treatment in those NSCLC patients with hyper progressive disease (138). Other suppressive targets, such as macrophage receptor with collagenous structure (MARCO), have also been described in TAM, and targeting them is expected to reverse the phenotype and curb cancers (139).

Chimeric antigen receptor macrophages (CAR-M) have drawn tremendous attention since they were introduced in 2020 (140). Given the tumor-homing ability of macrophages, CAR-M are expected to enter the solid tumor and exhibit antigen-specific-phagocytosis. They have been demonstrated to promote pro-inflammation, attract T cells, and prevent immunosuppression in TME (141). Similar to the evolution of CAR-T cell therapy, the cell sources of CAR-M have transitioned from using peripheral blood monocytes (140) to induced pluripotent stem cells (iPSC) (142), to now modifying *in situ* tumor macrophages to bypass cell isolation and tissue rejection issues (141). Additionally, CAR-M may potentially serve as a cargo to deliver drugs (143). CAR-M has shown an extraordinary ability to clear tumor cells in preclinical models as mentioned; however, how they interact with tissue-resident macrophages, whether CAR-M develop an innate memory response, whether trogocytosis occurs between CAR-M and other cells in the TME, and many other questions remain to be resolved.

## 5 Discussion

AMs are highly plastic immune cells that respond to environmental signals. NSCLCs associated with chronic inflammation and extrinsic stimuli, such as cigarette smoke and environmental pollutants, can transform TAMs phenotypes incapacitating their effector functions. Increasing evidence suggests that TRAMs are transcriptionally and epigenetically distinct from MoAMs, which can contribute to TAMs but exert different functions. These differences highlight the intricate relationships between myeloid cells and their environment. Discovering key regulatory factors that determine these disparities will provide depth to our understanding of the heterogeneity of macrophages and inform us on new lung cancer treatment options. Several tools will be beneficial in this pursuit. Genetic tracing can inform the origin of macrophages. Multi-omics approaches combined with pseudo-timing algorithms or temporal fluorescent labeling techniques can provide insights into the localization, interactions, and evolution between various cell types and dynamics in the disease states. *In vivo* murine tumor studies combining different therapies with targeting specific macrophage populations or regulators will inform basic biology as well as precision oncology. Given the abundance, tumor-homing ability, and plasticity of macrophages, targeting macrophages and macrophage cell therapy is desirable. Future treatments should be tailored based on the TME structure in each patient and logistically combine ICI to enhance efficacy.

## Author contributions

CC: Conceptualization, Visualization, Writing – original draft, Writing – review & editing. DA: Visualization, Writing – review & editing. DC: Writing – review & editing. FK: Funding acquisition, Resources, Supervision, Writing – review & editing.
