# Corrigendum: Alveolar macrophages in lung cancer: opportunities and challenges

**DOI:** 10.3389/fimmu.2023.1328927

**Published:** 2023-11-06

**Authors:** Cheng-Yen Chang, Dominique Armstrong, David B. Corry, Farrah Kheradmand

**Affiliations:** ^1^ Department of Medicine, Baylor College of Medicine, Houston, TX, United States; ^2^ Department of Pathology and Immunology, Baylor College of Medicine, Houston, TX, United States; ^3^ Biology of Inflammation Center, Baylor College of Medicine, Houston, TX, United States; ^4^ Center for Translational Research on Inflammatory Diseases, Michael E. DeBakey Department of Veterans Affairs Medical Center, Houston, TX, United States

**Keywords:** tissue-resident alveolar macrophages, smoking, lung cancer, immune checkpoint inhibitors, tumor-associated macrophages, tumor microenvironment

In the published article, there was an error in the article title. Instead of “*Alveolar Macrophages in Lung Cancer: Opportunities Challenges*”, it should be “*Alveolar Macrophages in Lung Cancer: Opportunities and Challenges*”.

The authors apologize for this error and state that this does not change the scientific conclusions of the article in any way. The original article has been updated.

